# Supplementary material for: Topological data analysis captures complex behavioral dynamics during naturalistic social interaction between domestic ferrets
Source: bioRxiv. 2026 Jul 7:2026.07.01.735818. Preprint. [Version 1] doi: 10.64898/2026.07.01.735818 (PMC13370394; doi:10.64898/2026.07.01.735818)
Supplement: Supplement 1 [file NIHPP2026.07.01.735818v1-supplement-1.pdf]

bioRxiv preprint doi: https://doi.org/10.64898/2026.07.01.735818; this version posted July 7, 2026. The copyright holder for this preprint (which was not certified by peer review) is the author/funder, who has granted bioRxiv a license to display the preprint in perpetuity. It is made available under a CC-BY 4.0 International license.

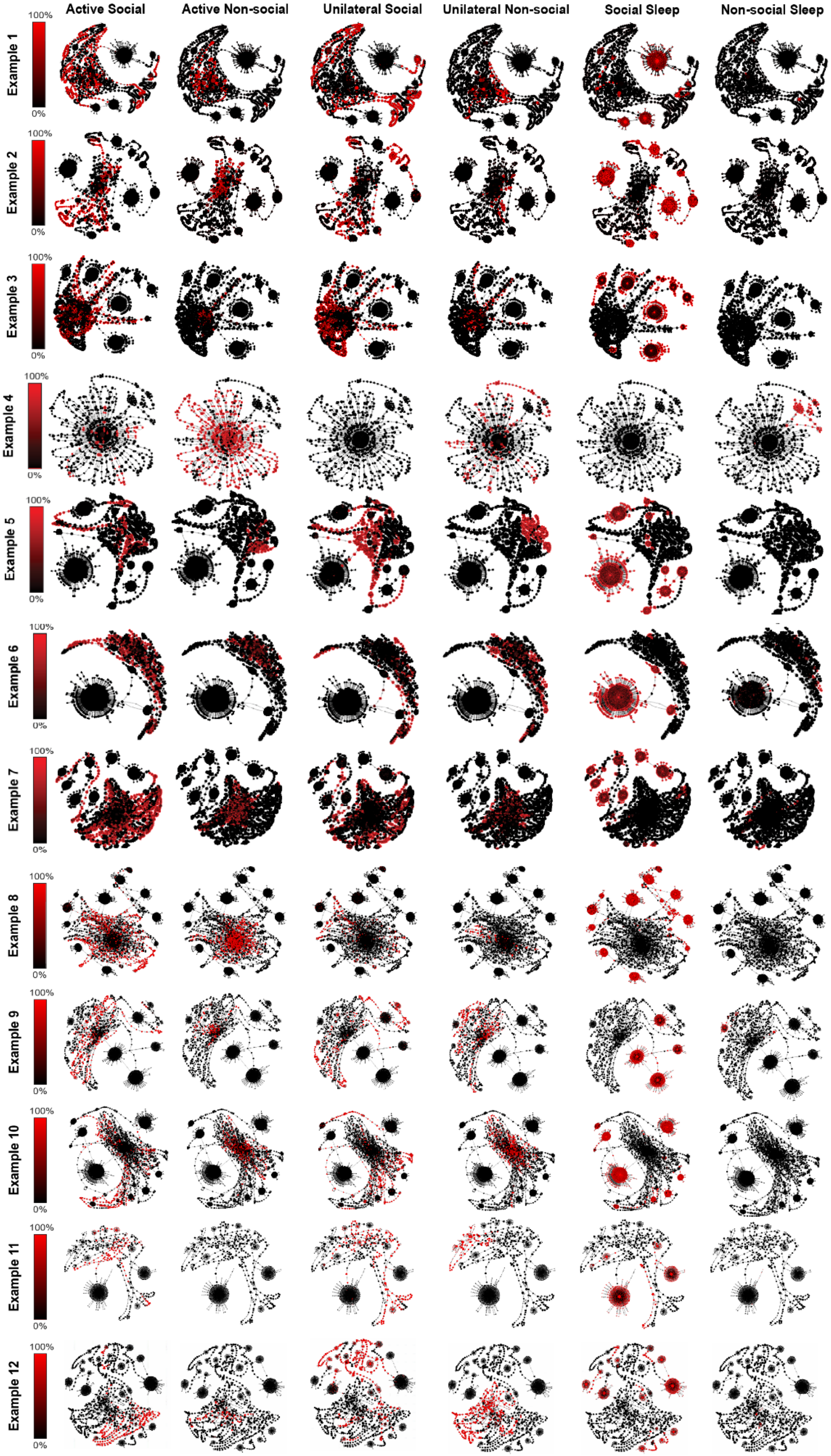

bioRxiv preprint doi: https://doi.org/10.64898/2026.07.01.735818; this version posted July 7, 2026. The copyright holder for this preprint (which was not certified by peer review) is the author/funder, who has granted bioRxiv a license to display the preprint in perpetuity. It is made available under a CC-BY 4.0 International license.

Supplemental Figure 1: Transition networks of each behavioral session. 12 behavioral sessions analyzed. The networks in each row have identical construction and color based on the indicated behavioral category for each column.

bioRxiv preprint doi: https://doi.org/10.64898/2026.07.01.735818; this version posted July 7, 2026. The copyright holder for this preprint (which was not certified by peer review) is the author/funder, who has granted bioRxiv a license to display the preprint in perpetuity. It is made available under a CC-BY 4.0 International license.

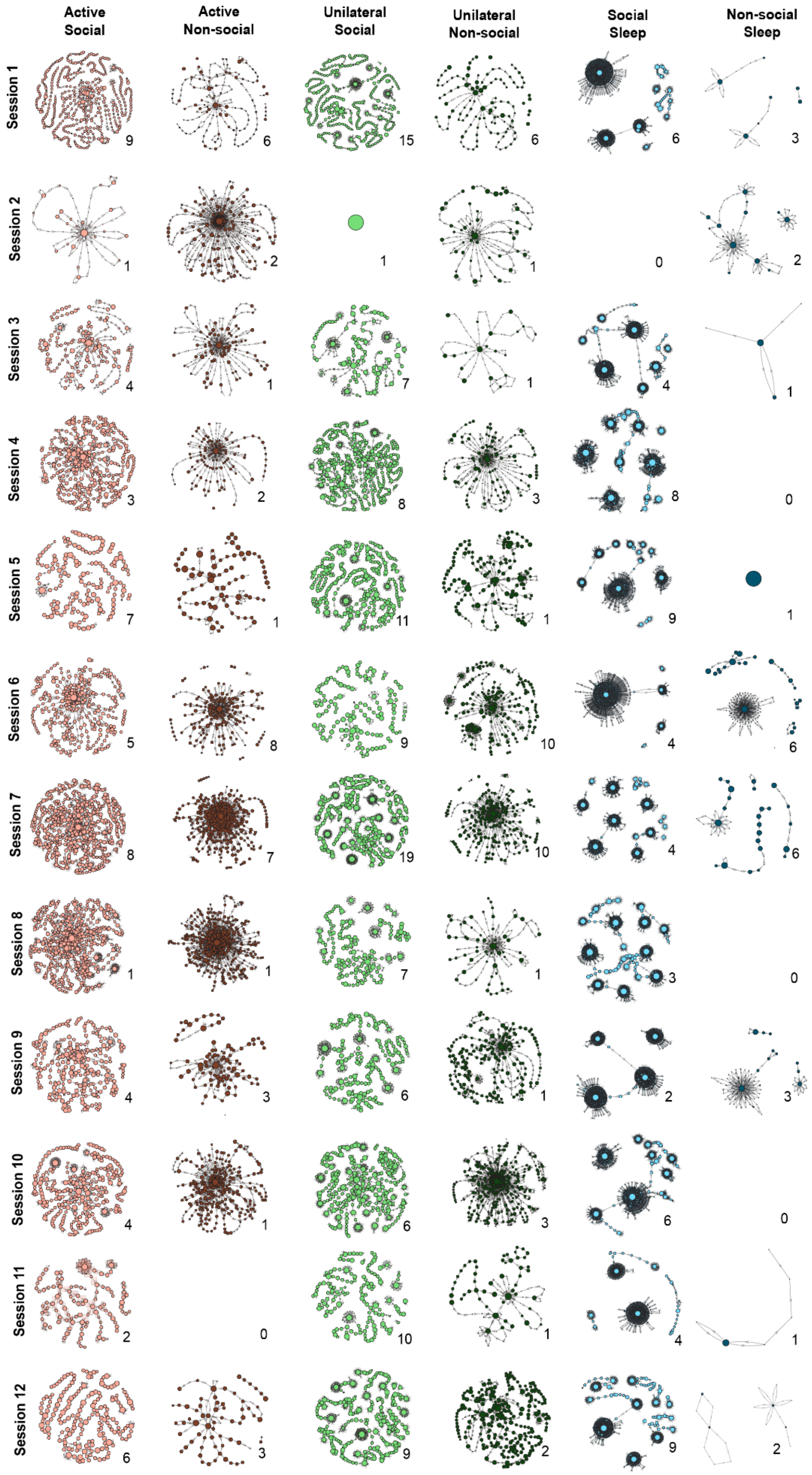

bioRxiv preprint doi: https://doi.org/10.64898/2026.07.01.735818; this version posted July 7, 2026. The copyright holder for this preprint (which was not certified by peer review) is the author/funder, who has granted bioRxiv a license to display the preprint in perpetuity. It is made available under a CC-BY 4.0 International license.

Supplemental Figure 2: Behavioral sub-networks computed from all 12 analyzed sessions. Number next to each sub-network indicates the number of connected components.

REFERENCES

1. Miller, J. H. & Page, S. E. *Complex Adaptive Systems: An Introduction to Computational Models of Social Life*. (Princeton University Press, 2007).
2. Bolis, D., Dumas, G. & Schilbach, L. Interpersonal attunement in social interactions: from *collective* psychophysiology to *inter- personalized* psychiatry and beyond. *Philos. Trans. R. Soc. B Biol. Sci.* **378**, 20210365 (2023).
3. Dumas, G., Lachat, F., Martinerie, J., Nadel, J. & George, N. From social behaviour to brain synchronization: Review and perspectives in hyperscanning. *IRBM* **32**, 48–53 (2011).
4. Moy, S. S. *et al.* Sociability and preference for social novelty in five inbred strains: an approach to assess autistic-like behavior in mice. *Genes Brain Behav.* **3**, 287–302 (2004).
5. Oliva, A., Fernández-Ruiz, A., Leroy, F. & Siegelbaum, S. A. Hippocampal CA2 sharp-wave ripples reactivate and promote social memory. *Nature* **587**, 264–269 (2020).
6. Gheusi, G., Bluthé, R.-M., Goodall, G. & Dantzer, R. Social and individual recognition in rodents: Methodological aspects and neurobiological bases. *Behav. Processes* **33**, 59–87 (1994).
7. Krueger-Burg, D. *et al.* The SocioBox: A Novel Paradigm to Assess Complex Social Recognition in Male Mice. *Front. Behav. Neurosci.* **10**, (2016).
